# Effects of a Syllable-Based Reading Intervention in Poor-Reading Fourth Graders

**DOI:** 10.3389/fpsyg.2017.01635

**Published:** 2017-09-20

**Authors:** Bettina Müller, Tobias Richter, Panagiotis Karageorgos, Sabine Krawietz, Marco Ennemoser

**Affiliations:** ^1^Department of Psychology IV, Educational Psychology, University of Würzburg Würzburg, Germany; ^2^Department of Psychology, University of Kassel Kassel, Germany; ^3^Department of Sport Science, Technical University of Darmstadt Darmstadt, Germany; ^4^Department of Special Education, Ludwigsburg University of Education Ludwigsburg, Germany

**Keywords:** older poor readers, primary school, word reading fluency, reading comprehension, syllable-based intervention

## Abstract

In transparent orthographies, persistent reading fluency difficulties are a major cause of poor reading skills in primary school. The purpose of the present study was to investigate effects of a syllable-based reading intervention on word reading fluency and reading comprehension among German-speaking poor readers in Grade 4. The 16-session intervention was based on analyzing the syllabic structure of words to strengthen the mental representations of syllables and words that consist of these syllables. The training materials were designed using the 500 most frequent syllables typically read by fourth graders. The 75 poor readers were randomly allocated to the treatment or the control group. Results indicate a significant and strong effect on the fluency of recognizing single words, whereas text-level reading comprehension was not significantly improved by the training. The specific treatment effect provides evidence that a short syllable-based approach works even in older poor readers at the end of primary school.

## Introduction

Recognizing written words and understanding written texts are among the most important competences children need to acquire in primary school. However, according to the Progress in International Literacy Study (PIRLS), 15.4% of fourth graders learning to read in German fail to master fundamental reading comprehension tasks (Bos et al., 2012). These students experience problems constructing a coherent representation of texts, because they are unable to connect information from different parts of the text or draw knowledge-based inferences that are crucial for comprehension. Importantly, most students with reading comprehension problems continue to struggle with basic reading processes at the word-level (Torppa et al., 2007), which leads to difficulties in understanding written texts at a level that is functional to meet the requirements of school and society. Individual differences in reading skills are known to be very stable across the school years. A longitudinal study with German children found that 70% of the children who fall behind in word reading fluency in Grade 1 still score low in reading fluency in Grade 8 (Landerl and Wimmer, 2008; see De Jong and Van der Leij, 2003 for similar results in a Dutch sample). A similar developmental pattern has been found for reading comprehension skills (Klicpera et al., 2006). Hence, targeted interventions are needed to remediate reading difficulties and to counter the negative developmental trajectories.

In this study, we examined a syllable-based word recognition intervention for German fourth graders with reading difficulties. We administered a 16-session training of basic word reading skills to these children and tested the effects of this training on the efficiency of written word recognition and the potential transfer effects on reading comprehension skills at the text level. We define the efficiency of word recognition as fast and accurate recognition of written words. As such, the efficiency of word recognition is an essential aspect of fluent reading and a prerequisite of good reading comprehension (Perfetti, 1985). In what follows, we will argue that a syllable-based intervention has a strong potential to foster poor readers’ reading skills. We begin with a discussion of reading interventions that work for struggling readers at the end of primary school. Then, we elaborate on the relevance of word reading fluency for reading comprehension and argue the benefits of focusing on syllables in word recognition training.

An ongoing debate persists as to which interventions are the most effective in fostering basic reading skills at the word level for struggling readers at the end of primary school. The most effective technique to train word recognition skills is phonics instruction, which is based on the alphabetic principle and aims at strengthening systematic associations between graphemes and phonemes. Several meta-analyses report small to medium effects of phonics programs on the accuracy of word decoding (*d* = 0.36 in Grade 6–12, Edmonds et al., 2009; *d* = 0.49 in Grade 2–6, Ehri et al., 2001; *d* = 0.26 in kindergarten to Grade 7, Suggate, 2014) or on aggregated outcome variables of accuracy, speed, and comprehension (*g* = 0.32 in children and adolescents, Galuschka et al., 2014). However, the effectiveness of phonics instruction seems to depend on grade level. According to a meta-analysis by Suggate (2010), the overall effect size (word reading and text comprehension) of phonics interventions (compared to other types of reading instruction) decreases during primary school education. Phonics instruction seems to work best for struggling readers in Grade 1 (Ehri et al., 2001). Starting at Grade 2, interventions focusing on comprehension become more effective. The basic idea of comprehension instruction is to develop self-regulated meaning making from texts using specific techniques such as question generation or summarizing (Edmonds et al., 2009). These interventions explicitly target on higher-order skills of integration and comprehension and waive teaching phonem-code strategies. Nevertheless, some studies have also found positive side-effects on accuracy and fluency of word reading (Suggate, 2014; Potocki et al., 2015; for effects on fluency, see Müller et al., 2015a).

Despite their potential in fostering reading fluency at the word level, comprehension interventions also require efficient word recognition skills to be effective (Müller et al., 2015b). Comprehension usually involves the teaching of resource-demanding reading strategies, which can be applied successfully only when children need to spend little cognitive effort for recognizing words (cf. lexical quality hypotheses, Perfetti and Hart, 2002). Children who learn to read a consistent language (such as German, Dutch, or Finnish) usually develop from a non-reader to an accurate decoder within the first school year. However, a broad and stable range in decoding fluency exists even in fourth graders whose word-reading accuracy is close to ceiling (cf. Landerl and Wimmer, 2008). A strong connection between fluency of word recognition and reading comprehension has been observed in students from first to fourth grade (*r* = 0.81 in a sample of Finnish first and second graders, Torppa et al., 2007; *r* = 0.67 in a sample of German third and fourth graders, Richter et al., 2017). Knoepke et al. (2014) investigated the relationship between visual word recognition processes (i.e., phonological recoding and orthographical decoding) and reading comprehension in German children in Grade 2 to Grade 4. In this study, the efficiency of both types of word recognition processes was a significant and unique predictor of reading comprehension, whereas their relative weight did not change across grade levels. That is, even in Grade 4 a strong relationship persisted between word recognition skills and reading comprehension. This relationship was strongest for orthographic decoding.

Achieving efficient orthographical decoding skills represents an important footstep in the development of reading fluency. Beginning readers rely primarily on phonological recoding, because most written words are unknown for them. More experienced readers, in contrast, read most words holistically via orthographical decoding by mapping (sub)lexical units or whole word forms directly on to their lexical entries (Frith, 1986; Ehri, 2005). However, some students miss this step in routinization and remain at the alphabetic stage of phonological letter-by-letter recoding. Research on dyslexic primary students in transparent orthographies from Grade 2 onward hint on large word length effects (i.e., impeding effects of the number of letters, Hautala et al., 2012) during word and non-word reading. These results indicate that these children still primarily rely on phonological recoding (Zoccolotti et al., 2005; Martens and de Jong, 2006; cf. Hautala et al., 2012). As a consequence, reading is slow and disfluent and associated with reading comprehension difficulties (Torppa et al., 2007). In sum, given the crucial importance of efficient orthographical decoding skills in reading development, constructing reading interventions targeted at developing these skills is strongly advisable.

According to theoretical models of reading development, children move from slow letter-by-letter decoding to the extraction of units that are larger than phonemes (cf. consolidated alphabetic phase, Ehri, 2005). Empirical studies suggest the importance of the syllable as the sublexical unit that bridges phonology and lexical entries (Hautala et al., 2012) through which word recognition fluency is facilitated. Colé et al. (1999) presented a target syllable followed by a word to French-speaking first graders. The participants’ task was to respond when the syllable appeared at the beginning of the word. At the end of Grade 1, only children with high scores in word reading fluency showed significant faster response times when the syllable matched the word. Disfluent children, in contrast, did not show a syllable compatibility effect. Häikiö et al. (2015, 2016) repeatedly found that marking syllables via hyphenation was beneficial for Finnish second graders with poor comprehension skills. In contrast, word recognition slows down significantly in children with good comprehension skills in the hyphenation condition, indicating that good readers already mastered reading with orthographic comparisons and probably accessed more than one syllable simultaneously (Grainger and Ziegler, 2011). Poor readers, in contrast, seem to experience difficulties in recoding larger chunks of letters. This observation is in accordance with results of Scheerer-Neumann (1981) who presented lists of pseudowords consisting of syllables that also appear in real German words. The pseudowords were presented either with or without graphical syllable segmentation. Poor readers achieved better results in the segmentation condition, whereas good readers showed the same accuracy in both conditions. These studies provide evidence for individual differences in syllable segmentation in beginning readers. Poor readers seem to experience difficulties in recoding syllabic units and reading holistically. Consequently, these readers’ word recognition is inefficient and uses a large amount of cognitive resources, which slows down reading.

In reading intervention studies, repeated reading of multi-letter consonant clusters (Hintikka et al., 2008; Huemer et al., 2008), of frequent syllables (Wentink et al., 1997; Bhattacharya and Ehri, 2004; Heikkilä et al., 2013), and of infrequent syllables (Huemer et al., 2010) has been shown to increase the accuracy and fluency of word recognition in children who had received at least 2 years of regular reading instruction. For transparent orthographies with clear syllabic structure, the effect in promoting reading speed is most pronounced for infrequent syllables (Finnish: Huemer et al., 2010; Heikkilä et al., 2013). In German, however, the syllabic structure is shallow and ambiguous (Seymour et al., 2003; Ziegler and Goswami, 2005) making it less easy for developing readers to extract syllabic units reliably. Thus, we assumed an intervention based on the most frequent syllables to be effective in promoting poor readers’ reading fluency. Given the consistency of German orthography, even poor readers are likely to acquire the competence to recognize German words accurately via phonological recoding, but their reading will be slow. An intervention based on extensive practice in syllable reading might help poor readers to learn the redundancy and the regularities of letters and words, which can be used for orthographic decoding (Ehri, 2005). We hypothesized that the mental representations of syllables and frequent words consisting of these syllables would be strengthened by the practice in reading materials based on the most frequent syllables. One syllable is contained in several words and can be presented in different positions of the word. Thus, the extensive practice of syllable recognition and segmentation to ameliorate word reading fluency among poor readers might be a promising intervention.

The aim of the current study was to examine effects of a newly developed syllable-based reading intervention for poor German-speaking readers in Grade 4. The theory behind syllabic reading predicts that the intervention should strengthen orthographic decoding processes as an indicator for word reading fluency and, indirectly, even promote reading comprehension (Perfetti and Hart, 2002). Another exploratory aim of the study was to investigate differences between poor and good reading fourth graders immediately after the intervention to assess the degree of improvement of poor readers compared to children without reading difficulties who receive no extracurricular reading training.

## Materials and Methods

### Design and Procedure

The study followed an experimental pre/post-test design with randomization at the class level. The data were collected as part of a longitudinal study that examined the effects of different reading interventions in primary school.

We first screened students with two standardized computer-based German-speaking reading tests. Subtests of the ProDi-L (Richter et al., 2012, 2017) were used to capture reading skills at the word level (phonological recoding, orthographic decoding, and access to word meanings) and a subtest of the ELFE (Lenhard and Schneider, 2006) to assess reading comprehension at the text level. We selected children with poor reading skills as participants. Poor reading skills were operationally defined as scores below percentile rank 50 on the class norms of both word recognition (mean composite score of the three ProDi-L subtests) and reading comprehension. Children were clustered in groups of four to six and the groups were randomly allocated at the class level to either the treatment or the control condition.

The nine groups in the treatment condition received the intervention between pre- and post-test. Student assistants (prospective teachers or psychology undergraduates) conducted the 16 treatment sessions, each session lasting 45 min. The training sessions occurred in addition to regular school curriculum twice a week. The efficiency of children’s reading processes was assessed again after the final training session with the ProDi-L, and reading comprehension was again assessed with the ELFE. The control condition was a wait-list group. Thus, the seven groups assigned to the control condition received a reading intervention after the post-test.

### Participants

The participating poor readers were 75 fourth graders from nine primary schools (23 classes). Of these, 43 children were allocated to the treatment condition and the remaining 32 to the control condition. The study was conducted in Giessen and Kassel (Germany). For organizational reasons, the treatment took place in Kassel and the control condition was divided between Giessen and Kassel. The average age of the participants was 10;13 years (*SD* = 1 year) and the proportion of boys and girls was nearly equal in both treatment condition, χ^2^ (1, *N* = 75) = 2.34, *ns* (see **Table 1**). The mean *Z*-values of the word recognition and reading comprehension skills at pretest were below the average in both treatment conditions compared to the class norms (see **Table 1**).

**Table 1:** Characteristics of the sample and mean Z-values of word recognition skills and reading comprehension at pretest by treatment condition (compared to class norms).

	Treatment group	Control group
*N*	43	32
Number of females	21	10
Word recognition (ProDi-L)		
Phonological recoding *M* (*SD*)	–1.11 (0.86)	–0.81 (0.99)
Orthographical decoding *M* (*SD*)	–1.31 (0.62)	–1.22 (0.72)
Access to word meaning *M* (*SD*)	–1.23 (0.60)	–1.12 (0.66)
Reading comprehension (ELFE) *M* (*SD*)	–0.96 (0.59)	–1.07 (0.64)

A group of 44 good readers (percentile rank above 50 on the class norms of both word recognition and reading comprehension, all from Kassel) was also tested at pre- and post-test to explore differences between poor and good readers at post-test on both outcome measures.

### Measured Variables

#### Assessment of Fluency of Word Recognition

We used a lexical decision task, the subtest orthographical decoding of the instrument ProDi-L (Richter et al., 2012, 2017), to assess the fluency of single-word reading. The children were required to decide whether a string of letters was a real word or a pseudoword. The 16 items, half of which were real German words and the other half pseudowords (orthographically and phonologically legal), varied systematically in length, frequency, and the number of orthographical neighbors. We used parallel versions of the subtest at pre- and post-test. The test recorded accuracy and latency of yes/no responses (provided with two response keys). An integrated test score was calculated as an indicator of the word recognition fluency. Therefore, a quotient of accuracy (mean number of correct responses) and response time (mean response time of the logarithmically transformed response times of all items when at least three items per scale had valid responses) was calculated. Thus, a high score indicates that a reader was faster and more accurate in word recognition fluency. The test–retest reliability was *r* = 0.25 (computed as the correlation of the pre- and post-test measures in the control group).

#### Measurement of Reading Comprehension

Reading comprehension skills were assessed with the subtest text comprehension of the ELFE 1–6 (Lenhard and Schneider, 2006). Children were presented with 20 short texts and were asked to answer questions concerning the content of each text by choosing one of four multiple-choice items. The test score is the sum of correct responses. The same 20 texts were presented at pre- and post-test in randomized order. The test–retest reliability was *r* = 0.55 (within the control group).

### Intervention: Syllable-Based Word Recognition Training

We designed the materials and the standardized manual of the intervention in cooperation with a learning therapist. All word materials were systematically selected based on the 500 most frequent German syllables in texts typically read by 9–12 year-old children (cf. data base childLex, Schroeder et al., 2015). The exercises included analyzing the syllabic structure of words by marking syllables with arcs during reading, finding the vowel nucleus within each syllable, combining prefixes and stems, and reading words aloud syllable-by-syllable. Special consideration was given to accurate phonological pronunciation of consonant clusters. Word recognition was first trained for single words with a regular spelling and a maximum length of four syllables. In advanced phases of the training, the complexity of materials increased to irregular words with up to eight syllables, and the materials included sentences and short texts. Several games were used to motivate children to read syllables and words as fast as they could, for instance, a kind of flash card reading (i.e., words were presented very briefly on cards in order to necessitate fast decoding, Wentink et al., 1997), games such as “syllable race” (i.e., moving a game character on a board according to the number of syllables in a word) and “syllable jump” (i.e., jumping on syllable cards on the floor in the order of the syllables in a word presented orally), or detecting two-syllabic words while showing cards with syllables on two stacks. The rationale behind the exercises was to strengthen the mental representations of syllables and orthographic representations that consist of these syllables, resulting in accuracy and speed improvement of word recognition.

The intervention was initially designed for 24 sessions. However, we found that after 3 weeks of intervention the exercises could be implemented faster than expected. Consequently, the number of sessions was reduced to 16 by combining two successive sessions within one.

## Results

### Data Analysis

Given the hierarchical structure of our data (students nested within classes nested within schools), we first estimated the intra-class correlations (ICCs) with random intercept multilevel models (Raudenbush and Bryk, 2002). The ICCs for the fluency of word recognition (ρ = 0.10) and reading comprehension (ρ = 0.52) indicated clustering effects in the data. Thus, we ran multilevel regression models with intercepts randomly varying between schools and classes. All models were estimated with the software package *lme4* for R (Bates et al., 2015). Significance level was set at 0.05, one-tailed (we tested directed hypotheses). Descriptive statistics and intercorrelations for all variables can be found in **Table 2**.

**Table 2:** Summary of intercorrelations, means, and standard deviations for all variables by treatment condition (treatment group below the diagonal, control group above the diagonal).

	1	2	3	4	*n*	*M*	*SD*
(1) Fluency of word recognition t1	—	0.25	0.49*	0.23	32	398.29	51.32
(2) Fluency of word recognition t2	–0.13	—	0.49*	0.32	30	414.35	76.86
(3) Reading comprehension t1	0.14	0.51^∗^	—	0.55*	32	6.94	2.83
(4) Reading comprehension t2	0.18	0.37^∗^	0.80**	—	28	8.04	4.56
*N*	43	37	43	37			
*M*	392.37	439.24	7.33	10.68			
*SD*	47.98	44.78	2.67	4.23			

*Fluency of word recognition = quotient of accuracy and reaction time, Range = 0–547.67 (Richter et al., 2012); Reading comprehension = sum of correct responses, Range = 0–20 (Lenhard and Schneider, 2006); t1 = pretest; t2 = post-test. Means and standard deviations for the treatment group (*N* = 43) are presented in the rows at the bottom of the table, for the control group (*N* = 32) in the columns on the right side of the table. ^∗^*p* < 0.05; ^∗∗^*p* < 0.01.*

Separate multilevel regression models were estimated for fluency of word recognition and reading comprehension measures with listwise deletion of missing data. The dummy-coded treatment condition (with the control group as reference category) was used as predictor, fluency and reading comprehension at post-test were used as outcome variables. The pretest score corresponding to the outcome variable was entered as a *z*-standardized covariate to control for pre-training differences. Given the significant pre-training difference in fluency of word recognition between participants in Kassel (*M* = 386.81, *SD* = 47.54) and Giessen (*M* = 427.23, *SD* = 43.12), *F*(1,73) = 8.98, *p* < 0.01, η^2^ = 0.11, the city where the data were collected was also entered as a dummy-coded predictor. All predictors were entered simultaneously into the models.

The visual inspection of standardized residuals versus unstandardized predicted values revealed that the assumptions of linearity, normality, and homoscedasticity were not violated in any of the models. The assumption of the independence of residuals was also supported (Cohen et al., 2003, Chap. 4 and 10). In the two models, two to three cases that deviated more than 2.5 standard deviations from the mean of the residuals were excluded from the analysis (Baayen, 2008, Chap. 7).

### Effects on Fluency of Word Recognition

**FIGURE 1:**
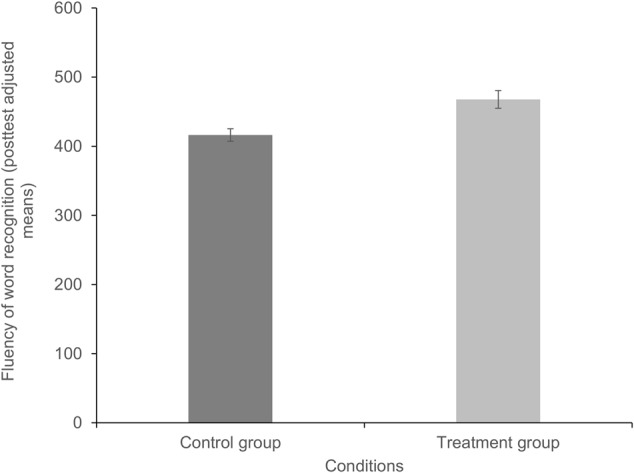
Comparison of the adjusted means for the fluency of word recognition per treatment condition at post-test. Higher values represent more efficient word recognition, i.e., more words were read accurately and fast.

The results for average effects of the syllable-based intervention on the poor readers’ reading skills compared to the control group are shown in **Table 3**. The multilevel regression revealed a significant treatment effect for word recognition fluency [β = 51.51, *t*(60) = 3.4, *p* < 0.05], which is illustrated in **Figure 1**. The effect size for the average treatment effect (computed as the difference between the adjusted means divided by the standard deviation of the outcome variable of the control group, cf. Mayer et al., 2014) indicated a strong effect (ES = 0.82). The adjusted means for the fluency of word recognition at post-test (estimated with the R-package *lmerTest*, Kuznetsova et al., 2015) were considerably higher in the treatment condition (*M_adjust_* = 467.76, *SD* = 43.35) than in the control condition (*M_adjust_* = 416.24, *SD* = 63.01).

**Table 3:** Fixed effects and variance components for the multilevel analyses with fluency of word recognition and reading comprehension as outcome variables, treatment condition as predictor, and corresponding pretest scores and city as covariates.

	Fluency of word recognition	Reading comprehension
Parameter	*B (SE)*	*B (SE)*
**Fixed effects**	
Intercept	446.79 (14.18)**	8.59 (1.85)*
Treatment (1) vs. Control Condition (0)	51.51 (15.17)*	1.96 (2.71)
Pretest score (*z*-standardized)	6.56 (6.72)	2.59 (0.31)**
City (0 = Giessen, 1 = Kassel)	–61.09 (20.21)*	–0.41 (2.90)
**Variance components**	
Classes within schools	0.00 (0.00)	0.00 (0.00)
Schools	0.00 (0.00)	9.04 (3.01)

*^∗^*p* < 0.05; ^∗∗^*p* < 0.01 (one-tailed).*

### Effects on Text-Based Reading Comprehension

**FIGURE 2:**
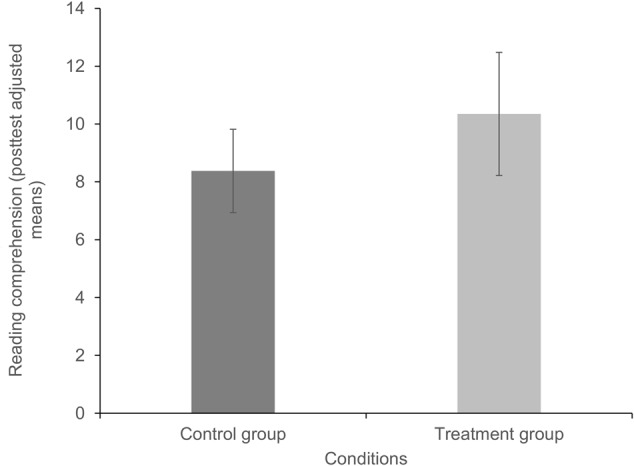
Comparison of the adjusted means for reading comprehension per treatment condition at post-test. Higher values represent a larger number of correct responses in a multiple-choice comprehension test (ELFE).

No significant effect was found between the treatment and the control group at post-test with reading comprehension as the outcome variable (see **Figure 2**). Although the reading comprehension scores in the treatment condition were higher than in the control condition, the comparison did not provide support for the transfer effects of the syllable-based reading intervention on reading comprehension at the text level. In this context, it is important to note that reading comprehension skills of the poor readers showed high stability from pre- to post-test, which might have prevented establishing a treatment effect.

### Comparison of Good and Poor Readers at Post-test

The estimates for the comparisons of the two groups of poor readers with the untreated good readers are shown in **Table 4**. The comparison between the treatment condition and the good readers revealed a significant negative effect on word recognition fluency [β = -49.61, *t*(74) = -2.88, *p* < 0.01], indicating that poor readers still differed significantly from good readers in fluency (*M* = 501.82, *SD* = 32.55) after the intervention. However, an even larger difference emerged for the poor readers in the control condition [β = -80.20, *t*(64) = -3.21, *p* < 0.01].

**Table 4:** Fixed effects and variance components for the multilevel analyses with fluency of word recognition and reading comprehension as outcome variables, treatment condition vs. untreated good readers as predictor, and corresponding pretest scores and city as covariates.

	Treatment group		Control group	
	Fluency of word recognition	Reading comprehension	Fluency of word recognition	Reading comprehension
Parameter	*B (SE)*	*B (SE)*	*B (SE)*	*B (SE)*
**Fixed effects**			
Intercept	494.77 (9.62)**	13.37 (0.73)**	547.22 (26.59)**	18.13 (2.29)**
Poor Readers (1) vs. Good Readers (0)	–49.61 (17.21)**	1.53 (1.20)	–80.20 (24.96)**	–6.47 (1.76)**
Pretest score (*z*-standardized)	9.41 (8.59)	4.77 (0.62)**	24.07 (10.28)*	2.02 (0.68)**
City (0 = Giessen, 1 = Kassel)			–60.07 (22.61)*	–2.55 (2.38)
**Variance components**			
Classes within schools	74.95 (8.66)	0.25 (0.50)	490.60 (22.15)	0.00 (0.00)
Schools	0.00 (0.00)	0.56 (0.75)	0.00 (0.00)	8.77 (2.96)

*The city was only included in the comparison of readers in the control condition and the good readers because the poor readers in the treatment condition and the good readers were all participants from Kassel. ^∗^*p* < 0.05; ^∗∗^*p* < 0.01 (two-tailed).*

The results for reading comprehension are somewhat surprising. There was no significant effect on the comparison of the treatment group with good readers [β = 1.53, *t*(70) = 1.28, *ns*]. Poor readers in the treatment group reached the same level of reading comprehension as good readers. In contrast, the comprehension of the poor readers in the control condition differed significantly from the good readers [β = -6.47, *t*(61) = -3.67, *p* < 0.01].

## Discussion

The aim of this study was to investigate the effects of a syllable-based reading intervention for poor readers in Grade 4. We assumed that the intervention would lead to gains in word reading fluency and reading comprehension.

The results of this study support primarily the first prediction. The syllable-based training had a significant and strong effect on the fluency of recognizing single words. The effect was similar in size compared to earlier studies on syllable reading interventions in orthographies with unambiguous syllable boundaries (Huemer et al., 2010). This result is encouraging given the complex syllabic structure in German. To the best of our knowledge, only one other intervention study with German-speaking primary school children investigated a training based on syllables as the unit of analysis (Ritter, 2010). In this study, a small group (*N* = 48) of third and fourth graders with poor reading skills was divided into a treatment group, placebo control group, and wait-list control group. The treatment group received an 18-session training of syllable and morpheme segmentation in one-to-one sessions. The results hint on improvements in word reading fluency and accuracy. The treatment effect of our study strengthens and expands these results. Our material was designed systematically based on the 500 most frequent written syllables for 9 to 12 year-olds, and the focus was on reading those syllables (i.e., frequent words consisting of these syllables on different positions). The intervention was implemented in small groups with a heterogeneous sample of relatively poor readers. We found that poor readers in the treatment condition outperformed same-skilled children in the control group at the post-test in reading fluency and that their deficits compared to the good readers’ reading fluency was reduced. These results are promising. One explanation for the strong effect size might be the composition of the sample of poor readers who received the treatment in our study. Meta-analytic results suggest that children with less severe reading impairments (operationalized as below-average readers) experience greater gains from reading interventions compared to children with severe deficits (operationalized as more than 1 standard deviation below the average; Suggate, 2010; Heikkilä et al., 2013; Galuschka et al., 2014).

In contrast to the fluency of word recognition, text-level reading comprehension was not significantly improved by the training. The result that a syllable-based training only led to specific improvements in word reading skills but lack effects on comprehension scores with older poor readers was already found in other samples, for example with struggling 13 year-old French readers (Potocki et al., 2015). Interestingly, most of the previous studies evaluating syllabic reading interventions did not examine effects on reading comprehension (Wentink et al., 1997; Huemer et al., 2010; Heikkilä et al., 2013). An intervention study by McCandliss et al. (2003) suggests that reading comprehension of poor readers above Grade 1 benefits from word recognition training if word reading is embedded in texts and if the participating children are repeatedly asked to elaborate on the meaning of those texts. Although words were read in the context of shorts texts in the second half of the intervention presented here, comprehension was not in the focus of the training. For example, exercises involving word meanings or comprehension of words in their linguistic contexts were not included in the training, explaining the lack of effects on comprehension. Furthermore, a longitudinal study with struggling Finnish-speaking readers in Grade 1 and 2, Torppa et al. (2007) found that reading comprehension developed rapidly only after the word recognition skills had reached a sufficient level (cf. Juul et al., 2014). Thus, it seems worthwhile to investigate which level of fluent syllable reading is required to achieve transfer effects on higher-order comprehension.

Another interpretation of the results for reading comprehension is fed by the observation that comprehension scores of poor readers who received the training did not differ significantly from those of the good readers in the sample. In contrast, the post-test reading comprehension scores of the poor readers in the control group still differed significantly from the good readers. This pattern of results suggests that the syllable-based training might indeed have had a positive effect on comprehension. The lack of a significant effect compared to the control group might have been due to a lack of power that was not sufficient to detect small to medium treatment effects.

Despite these open questions, the result of the specific improvement in word reading fluency among German-speaking fourth graders with poor word recognition and reading comprehension skills is encouraging against the background of the persistence of individual differences in reading fluency (De Jong and Van der Leij, 2003; Landerl and Wimmer, 2008) and the relevance of fluent reading for sufficient reading skills in transparent orthographies. A short, but focused intervention making frequent syllables salient as a unit of recognition seems to have the potential to raise the fluency of word recognitions even in poor readers who have already received at least 3 years of regular reading instruction.

## Ethics Statement

This study was carried out in accordance with the recommendations of “Ministry of Education and Cultural Affairs, Hesse, Germany (Hessisches Kultusministerium)” with written informed consent from all subjects. The parents of all subjects gave written informed consent in accordance with the Declaration of Helsinki. Principals of the schools participating in the study gave written consent after the school conference (i.e., the majority of teachers agreed to realize the study in their school).

The protocol was approved by the “Ministry of Education and Cultural Affairs, Hesse” (cf. Education Act of Hesse, section 84).

## Author Contributions

The study reported here was realized in cooperation between the authors. The material and manual of the intervention were designed by BM and TR in cooperation with a learning therapist. Data collection took place in Kassel (organized by BM and TR) and Giessen (organized by SK and ME). Statistical analyses were done by BM, PK, and TR.

## Conflict of Interest Statement

The authors declare that the research was conducted in the absence of any commercial or financial relationships that could be construed as a potential conflict of interest.
